# Sugar metabolism, chip color, invertase activity, and gene expression during long-term cold storage of potato (*Solanum tuberosum*) tubers from wild-type and vacuolar invertase silencing lines of Katahdin

**DOI:** 10.1186/1756-0500-7-801

**Published:** 2014-11-16

**Authors:** Amy E Wiberley-Bradford, James S Busse, Jiming Jiang, Paul C Bethke

**Affiliations:** United States Department of Agriculture – Agricultural Research Service, Vegetable Crops Research Unit, 1575 Linden Dr., Madison, WI 53706 USA; Department of Horticulture, University of Wisconsin-Madison, 1575 Linden Dr., Madison, WI 53706 USA

**Keywords:** Vacuolar invertase, Potato, Sugar metabolism, Cold storage, Starch synthase, Sucrose synthase, Invertase inhibitor, ADP-glucose pyrophosphorylase, Potato chip processing quality

## Abstract

**Background:**

Storing potato tubers at low temperatures minimizes sprouting and disease but can cause an accumulation of reducing sugars in a process called cold-induced sweetening. Tubers with increased amounts of reducing sugars produce dark-colored, bitter-tasting fried products with elevated amounts of acrylamide, a possible carcinogen. Vacuolar invertase (VInv), which converts sucrose produced by starch breakdown to glucose and fructose, is the key determinant of reducing sugar accumulation during cold-induced sweetening. In this study, wild-type tubers and tubers in which *VInv* expression was reduced by RNA interference were used to investigate time- and temperature-dependent changes in sugar contents, chip color, and expression of *VInv* and other genes involved in starch metabolism in tubers during long-term cold storage.

**Results:**

VInv activities and tuber reducing sugar contents were much lower, and tuber sucrose contents were much higher, in transgenic than in wild-type tubers stored at 3-9°C for up to eight months. Large differences in *VInv* mRNA accumulation were not observed at later times in storage, especially at temperatures below 9°C, so differences in invertase activity were likely established early in the storage period and maintained by stability of the invertase protein. Sugar contents, chip color, and expression of several of the studied genes, including AGPase and GBSS, were affected by storage temperature in both wild-type and transgenic tubers. Though transcript accumulation for other sugar-metabolism genes was affected by storage temperature and duration, it was essentially unaffected by invertase silencing and altered sugar contents. Differences in stem- and bud-end sugar contents in wild-type and transgenic tubers suggested different compartmentalization of sucrose at the two ends of stored tubers.

**Conclusions:**

*VInv* silencing significantly reduced cold-induced sweetening in stored potato tubers, likely by means of differential *VInv* expression early in storage. Transgenic tubers retained sensitivity to storage temperature, and accumulated greater amounts of sucrose, glucose and fructose at 3°C than at 7-9°C. At each storage temperature, suppression of *VInv* expression and large differences in tuber sugar contents had no effect on expression of AGPase and GBSS, genes involved in starch metabolism, suggesting that transcription of these genes is not regulated by tuber sugar content.

## Background

Cultivated potato (*Solanum tuberosum* L.) is one of the most important food crops in the world. In the United States, over half of the potato tubers produced are processed to make chips, french fries, and similar products [1]. A crucial quality requirement for these tubers is the ability to produce light-colored, flavorful fried products that will be acceptable to consumers. Color and taste depend largely on the abundance of reducing sugars, primarily glucose and fructose, in raw tubers. During frying, these reducing sugars react with free amino acids in a nonenzymatic Maillard reaction that results in dark-colored, bitter-tasting products [2, 3]. Another product of the Maillard reaction is acrylamide, a neurotoxin and suspected carcinogen [4, 5]. Potato chips and fries have particularly high acrylamide contents compared to other processed foods [6], and it has been suggested that decreasing the reducing sugar content in tubers is a highly effective way to minimize acrylamide production in potato products [7, 8]. Up to 20% of the potatoes intended for processing in the United States are rejected because their reducing sugar contents are too high [9].

Potatoes stored at temperatures below approximately 10°C accumulate more sucrose than those stored at higher temperatures, and some of this sucrose is converted to glucose and fructose by the vacuolar enzyme acid invertase (VInv). This process of sugar accumulation is called cold-induced sweetening (CIS) [10]. Tubers intended for fry and chip processing are typically stored at 8-10°C to minimize reducing sugar formation and subsequent browning of fried products. Unfortunately, storage at these relatively warm temperatures dramatically increases rates of sprouting and spoilage from disease compared with storage at 3-5°C. It would therefore be highly advantageous to develop potato cultivars that could be stored at low temperatures without undergoing CIS.

The enzymes involved in the conversion of starch to sucrose and reducing sugars during CIS have been identified, and most have been characterized at molecular and biochemical levels [3, 10, 11]. During CIS, the net rate of sucrose synthesis increases [12]; some of this sucrose is transported to the vacuole, where VInv converts it to glucose and fructose. Accumulation of reducing sugars during cold storage is strongly linked to VInv activity [13, 14]. Transcription of *VInv* is rapidly up-regulated at temperatures that promote CIS [14–16]. For tubers of cv. Desirée that were stored at 4°C, *VInv* expression was up-regulated within the first seven days of cold storage, though this was followed by a large, gradual reduction in transcript amount that occurred over 16 weeks [15]. Changes in *VInv* expression during long-term cold storage have rarely been examined in detail, and their relationship to the expression of other genes involved in starch metabolism has not been studied previously.

Several reports have shown that genetic manipulation of VInv can reduce CIS. For example, increased expression of an invertase inhibitor from tobacco was found to decrease formation of reducing sugars in cold-stored potato tubers [17]. Likewise, transcripts for endogenous invertase inhibitors were more abundant in cultivars resistant to CIS than in susceptible cultivars [18]. Use of antisense inhibition decreased VInv activity in cold-stored tubers of cv. Desirée by up to 92% and reduced tuber hexose contents by 43% [15]. RNA interference (RNAi) suppression of *VInv* expression decreased transcript accumulation by up to 78% and activity by 68% in cv. N2 [19] and reduced acrylamide in lines of Ranger Russet by almost 90% [20]. In lines of MegaChip, Dakota Pearl, and Atlantic in which *VInv* was silenced by RNAi, tuber reducing sugar contents decreased and chip color became lighter as the extent of gene silencing increased [21]. Russet Burbank lines with RNAi suppression of *VInv* had considerably less reducing sugar accumulation than untransformed tubers through five months of storage at 8°C [22]. In those lines, *VInv* silencing was less effective at the tuber stem (basal) end than at the tuber bud (apical) end. Furthermore, changes in VInv activity during storage in wild-type (WT) and RNAi lines of Russet Burbank were consistent with changes in *VInv* expression at the tuber stem end but not at the tuber bud end. The authors suggested that changes in *VInv* message do not result in changes in VInv activity when *VInv* mRNA amounts are relatively small [22]. RNAi suppression of *VInv* in cv. Katahdin tubers almost completely prevented increases in reducing sugar contents when tubers were stored for up to 180 days at 4°C, and chips made from these tubers had acceptable color and less than one-tenth the acrylamide of untransformed Katahdin tubers [16]. *VInv* silencing was highly effective in three of these Katahdin lines, with 97-99% reduction in *VInv* mRNA 14 d after harvest. Data for *VInv* expression in RNAi and WT tubers at later times were not available, differences in expression and activity along the tuber axis were not reported, and effects of *VInv* suppression and related sugar accumulation on expression of other genes involved in starch metabolism were not investigated. In addition, previous *VInv* silencing studies have compared tubers in one high and one low storage temperature, rather than across a range of storage temperatures, which is necessary to determine the extent to which *VInv* expression regulates the temperature sensitivity of sugar metabolism in stored tubers.

In the present study, molecular and biochemical changes associated with long-term cold storage of tubers from the three most highly silenced of these Katahdin lines (Line 1, Line 2 and Line 3) [16] were investigated in greater detail. Tubers were stored at various temperatures in order to determine whether the effectiveness of *VInv* suppression varied with storage temperature and time in storage, and to determine whether the temperature sensitivity of tubers in which *VInv* was silenced differed from that of wild-type tubers. Time- and temperature-dependent changes in expression of genes for additional components of pathways of sugar metabolism were investigated to ascertain whether the sugars produced by VInv activity affected expression of these genes, and whether they might be suitable targets for further genetic manipulation to improve tuber quality. Spatial differences in gene expression and sugar concentration were determined by sampling tissue at the stem and bud ends of tubers. The two ends of tubers have often been found to have different sugar contents, and differences in activity of a few enzymes, including VInv and sucrose synthase, have been reported [23–25]. All samples were collected 3 to 8 months after harvest in order to uncover molecular and biochemical changes associated with long-term cold storage.

## Methods

### Plant materials; growth and storage conditions

Tubers of wild-type Katahdin [26] and RNAi lines 1, 2, and 3 of Katahdin [16] were planted at the University of Wisconsin Hancock Agricultural Research Station in Hancock, Wisconsin, during the 2010 growing season. All necessary regulatory approvals for field growth of transgenic lines were obtained from the U.S. Department of Agriculture Animal and Plant Health Inspection Service before planting. Seed tubers were planted in a randomized complete block design with four blocks and two 5-hill replicates of each line and controls within each block. Standard cultivation and management practices were employed during the growing season. Tubers were mechanically harvested on October 1, 2010 and stored at 13°C to promote final skin set and formation of wound periderm. Tubers were treated with isopropyl N-(3-chlorophenyl)carbamate (CIPC) on November 1, 2010 to prevent sprouting; on November 11, the tubers of each line were randomized into four treatment groups with final storage temperature set points of 3, 5, 7, or 9°C. Cooling of storage lockers began on November 30 at a maximum rate of 0.06°C per 8 hours, a typical cooling rate used in commercial potato storage facilities. All lockers reached final temperature set points by the fourth week of January 2011. At that time, and at six-week intervals thereafter, twelve tubers per line per storage temperature were selected at random for biochemical, molecular and chip color analysis.

### Chipping analysis

Washed, unpeeled tubers were cut in half lengthwise and three, 1-mm-thick slices were cut from one half for frying. Slices were fried in cottonseed oil at 188°C for 2 min 10 s. Fried chips were crushed for color analysis using a D25LT colorimeter (HunterLab, Reston, VA). Each sample consisted of 18 crushed chips, three chips from each of six tubers per line per storage temperature. Unpeeled chips with Hunter scores of 50–55 or higher may be acceptable to the potato processing industry [27, 28].

### Sugar, protein, and mRNA extraction and analysis

At each sampling time, three tissue samples from the stem (basal) ends and three tissue samples from the bud (apical) ends of six tubers per line per storage temperature were collected, frozen in liquid nitrogen, and stored at −80°C until use. Each sample was a cylinder of tuber tissue with a fresh weight of approximately 0.5 g. One tissue sample from each end of each tuber was used for sucrose, glucose, and fructose quantification using high-performance liquid chromatography as described by Bethke et al. [29]. From the remaining two tissue samples, one from each end of each tuber was used for RNA analysis and the other was used for protein extraction. For each line, samples for RNA analysis were separated into two groups of three bud end samples and two groups of three stem end samples, and each pooled sample of three was ground under liquid nitrogen using a freezer mill (model 6770, SPEX SamplePrep, Metuchen, NJ) to make two pooled tissue samples for each tuber end of each line at each storage temperature and sampling time. Pooled samples for protein extraction were prepared using the same procedure. Ground samples were stored at −80°C.

Protein extraction and desalting were carried out as described by Bhaskar et al. [16], with about 0.5 g of pooled, ground tissue and 2 mL of extraction buffer. Protein concentrations in these extracts were determined using the Bio-Rad protein assay reagent (Bio-Rad Laboratories, Hercules, CA). Protein extracts were assayed for acid invertase activity as described by Bhaskar et al. [16], with all samples assayed in triplicate and activities expressed in nmol glucose min^−1^ mg^−1^ protein. Both extracts that had been vortexed to inactivate endogenous invertase inhibitors and non-vortexed extracts were assayed.

RNA was extracted from pooled, ground tissue samples using the Agilent Plant RNA Isolation Mini Kit (Agilent Technologies, Inc., Santa Clara, CA) according to the manufacturer’s instructions. Twenty microliters of extraction solution were used per milligram of tissue. RNA integrity was checked visually using agarose gel electrophoresis, and purity was evaluated using a NanoDrop 1000 (NanoDrop Products, Wilmington, DE) with absorbances measured at 230, 260, and 280 nm. RNA was DNase-treated using a DNase Treatment Kit (Ambion, Austin, TX) according to the manufacturer’s instructions. Reverse transcription was carried out on 800 ng of total RNA using the SuperScript III kit (Invitrogen Corporation, Carlsbad, CA) according to the manufacturer’s instructions. After reactions were completed, nuclease-free water was added to each to bring the volume to 65 μL. Two microliters of this mixture (containing 25 ng of total RNA) were used as template for each quantitative PCR. For each gene, only a single product was formed and calculated PCR efficiencies were between 90% and 105%.

Quantitative PCR was carried out using a BioRad iCycler with Maxima SYBR Green/Fluorescein qPCR Master Mix (Fermentas Inc., Glen Burnie, MD). The thermal profile consisted of one cycle of 95°C for 10 min, followed by 40 cycles of 95°C for 15 s, 55°C for 30 s, and 72°C for 30 s. A melt curve was then collected with temperatures from 55 to 95°C. Fluorescence data were collected during the extension phase and the melt curve. Reactions for all genes in each sample were carried out in triplicate. Primer sequences and final concentrations are listed in Table 1.

**Table 1:** **Sequences and concentrations of primers used for quantitative PCR**

Gene (Accession)	Forward primer, concentration	Reverse primer, concentration
Actin 97 (TC164213)	atgttcccgggtattgctgacaga, 0.4	ctgcctttgcaatccacatctgct, 0.4
AGPase (AY186620)	ggagtccgattcaatgtgagaagaag, 0.4	ccaaaacactccggctagcatc, 0.4
GBSS (EU403426)	tacacaagagtggaacccagcgac, 0.4	tgtcaacaggcaagccaactgc, 0.4
INH (FJ810207)	caccctacaatccgatccacgta, 0.4	tcgccacgtaacactggctaagt, 0.4
SPS (BQ510597)	tccacaggtcgcaagagtatcagg, 0.8	ccggataaaacacttcgctcccac, 0.2
SuSy (AJ537575)	tttgaggcctggtgtctgggaataca, 0.4	tccattcgaggctccgtcgacaa, 0.4
VInv (TC163068)	aaacgggttggacacatcat, 0.2	aacccaattccacaatccaa, 0.2

All sequences are given 5′-3′, and concentrations are μM. *Abbreviations*: *AGPase* ADP-glucose pyrophosphorylase small subunit, *GBSS* granule-bound starch synthase, *INH* invertase inhibitor 3, *SPS* sucrose phosphate synthase 2, *SuSy* sucrose synthase 4, *VInv* vacuolar acid invertase. Accessions for Actin 97 and VInv are from the Gene Index Project; all others are from GenBank.

## Results

### Sugar contents of stored tubers

**Figure 1:**
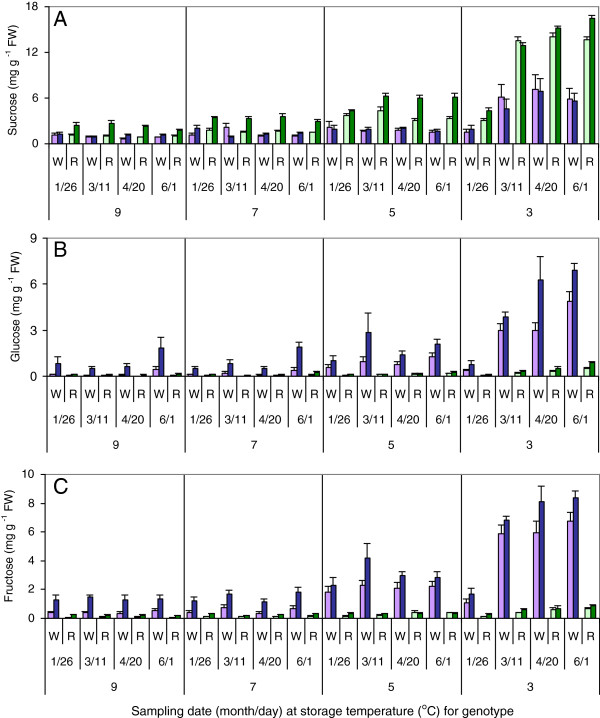
**Tuber sugar contents.** Sugar concentrations in WT (W) and RNAi (R) tubers with suppressed *VInv* expression stored for different lengths of time at 3, 5, 7, or 9°C. **(A)** Sucrose; **(B)** Glucose; **(C)** Fructose. Light purple bars represent the bud ends of WT tubers; dark purple bars, the stem ends of WT tubers; light green bars, the bud ends of RNAi tubers; and dark green bars, the stem ends of RNAi tubers. Bars show averages of samples from six (WT) or eighteen (RNAi) different tubers ± standard error. Tubers were harvested on October 1, 2010 and sampled in 2011.

Tubers from the invertase silencing lines (RNAi tubers) had higher amounts of sucrose and lower amounts of glucose and fructose than untransformed controls (WT tubers) from January through June (Figure 1). These differences became larger as storage temperature decreased. All three RNAi lines had similar sugar contents, so their results were averaged to simplify presentation of the data.

Sucrose contents in tubers stored at 7 and 9°C averaged 1.2 mg g^−1^ fresh weight (FW) at both ends of WT tubers, but the bud and stem ends of RNAi tubers at these temperatures had average sucrose contents of 1.3 and 2.8 mg g^−1^ FW, respectively (Figure 1A). Tuber sucrose content was higher at 5°C than at 7 and 9°C for both WT and RNAi lines, and higher still at 3°C. Tubers stored at 3°C reached their final storage temperature in January, and beginning with the March sampling, a dramatic increase in sucrose contents occurred, with values rising to 6–7 mg g^−1^ FW in WT and 14 mg g^−1^ FW in RNAi tubers. Sucrose contents in the bud and stem ends of WT tubers were similar at all temperatures and storage durations, but were much higher in the stem ends than the bud ends of RNAi tubers at 5, 7, and 9°C. The average ratios of tuber stem-end to bud-end sucrose in WT and RNAi tubers were 1.2 and 2.2, respectively, at these temperatures, and these values differed at the p < 0.05 level.

WT tubers stored at 7 and 9°C contained about 0.2 mg glucose and 0.5 mg fructose g^−1^ FW in the bud end and about 0.9 mg glucose and 1.5 mg fructose g^−1^ FW in the stem end, while RNAi tubers stored at these temperatures had 10-20% of these amounts (Figure 1B,C). At all sampling times, WT tubers stored at 5°C had higher glucose and fructose contents than those stored at 7 or 9°C. The amounts of these reducing sugars in RNAi tubers were much lower than in WT tubers at all times and temperatures, but were significantly (p < 0.05) higher at 5°C than at 7 or 9°C in samples collected between March and June. WT tubers stored at 3°C had a dramatic increase in reducing sugar content that was first observed in March. Reducing sugar contents eventually reached 5 mg g^−1^ FW at the bud ends and 7 mg g^−1^ FW at the stem ends of these tubers. Glucose and fructose contents in RNAi lines stored at the same temperature were approximately 10% of these amounts. Glucose and fructose contents were higher in the stem ends than the bud ends of WT tubers but were similar to each other at the two ends of RNAi tubers.

The ratio of glucose to fructose was significantly higher in the stem ends than in the bud ends of WT tubers at almost all times and temperatures (p < 0.05), but it was similar in the two ends of RNAi tubers stored through April (Table 2). Glucose/fructose ratios were higher in WT and RNAi tubers at all temperatures in June than at the same temperatures at earlier sampling times (p < 0.01 according to Student’s t-test; data not shown).

**Table 2:** **Ratios of glucose to fructose in bud (B) and stem (S) ends of WT and RNAi tubers**

	WT		RNAi	
Temperature (°C)	B	S	B	S
9	0.21 + 0.01	0.42 ± 0.05	0.45 ± 0.05	0.39 ± 0.03
7	0.28 + 0.02	0.43 ± 0.06	0.34 ± 0.03	0.35 ± 0.02
5	0.34 ± 0.04	0.50 ± 0.06	0.41 ± 0.03	0.39 ± 0.03
3	0.45 ± 0.03	0.55 ± 0.05	0.48 ± 0.02	0.50 ± 0.03

Values are averages ± standard error of 18 (WT) or 54 (RNAi) samples collected in January through April. Tubers were harvested on October 1, 2010 and sampled in 2011.

### Chip quality from stored tubers

**Figure 2:**
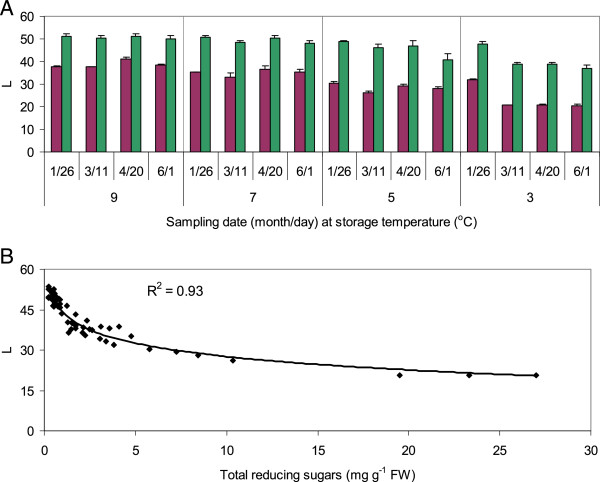
**Chip colors and correlation with sugar contents.** Average L-values of chips **(A)** and correlation of L-values with reducing sugar contents **(B)** from tubers stored through June at 3, 5, 7, or 9°C. Purple bars represent WT tubers; green bars, RNAi tubers. Values are the averages of two samples of WT and six samples of RNAi lines ± standard error; each sample is comprised of three chips from each of six tubers. Tubers were harvested on October 1, 2010 and sampled in 2011. Higher L-values correspond to lighter chip color.

All RNAi tubers yielded paler chips, with higher luminosity (L) values, than WT tubers, regardless of storage temperature or duration (Figure 2A). Color values for the three RNAi lines were not significantly different from each other, so their results were averaged. Chip color did not change significantly for WT and RNAi tubers stored at 9 or 7°C between January and June. Chips from WT tubers, but not RNAi tubers, stored at 7°C were slightly darker than those stored at 9°C. Chip color L-values from January through June averaged 37.8 ± 1.6 and 35.0 ± 1.4 for samples made from WT tubers stored at 9 and 7°C, respectively, and 50.7 ± 0.6 and 49.6 ± 1.3 for samples made from RNAi tubers. Chip color L-values decreased with decreasing storage temperature and at 5°C were 28.6 ± 0.9 and 45.7 ± 1.7 for WT and RNAi tubers, respectively. Tubers stored at 3°C later than March yielded the darkest chips, with average L-values of 20.7 ± 0.1 and 38.3 ± 0.6 for WT and RNAi tubers, respectively. Chip color was correlated with average tuber reducing sugar content (Figure 2B).

### Acid invertase activity in stored tubers

**Figure 3:**
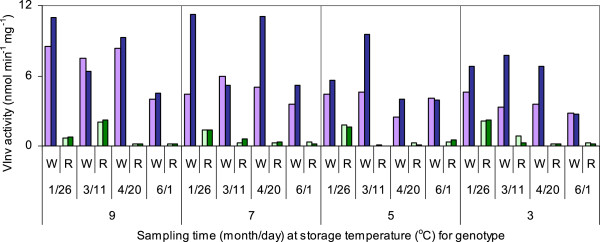
**Tuber acid invertase activities.** Average acid invertase (Inv) activities in extracts from WT (W) and RNAi (R) tubers stored for different lengths of time at different temperatures. Light purple bars represent the bud ends of WT tubers; dark purple bars, the stem ends of WT tubers; light green bars, the bud ends of RNAi tubers; and dark green bars, the stem ends of RNAi tubers. Values are the averages of two (WT) or six (RNAi) independent samples ± standard error. Tubers were harvested on October 1, 2010 and sampled in 2011.

Acid invertase activity in the bud ends of WT tubers was seven times that in the bud ends of RNAi tubers; in the stem ends of WT tubers, it was ten times that in the stem ends of RNAi tubers when averaged across all sample times and temperatures (Figure 3). Activities were similar in the three RNAi lines, so these values were averaged. Activities of non-vortexed extracts were similar to those of extracts that had been vortexed to inactivate endogenous invertase inhibitors; only the latter data are shown. The average difference between vortexed and non-vortexed activities was 0.23 ± 0.05 nmol min^−1^ mg^−1^. Invertase activities at the bud ends of WT tubers decreased with decreasing temperature at most sampling times (p < 0.05 in 2-way ANOVA), though activities at the stem ends were more similar across temperatures. In WT tubers, acid invertase activity in tuber stem-end tissue was greater than that in bud-end tissue when averaged across all sampling times and temperatures (p < 0.05), while in RNAi tubers, activities at the two ends of the tubers did not differ.

### mRNA accumulation in stored tubers

**Figure 4:**
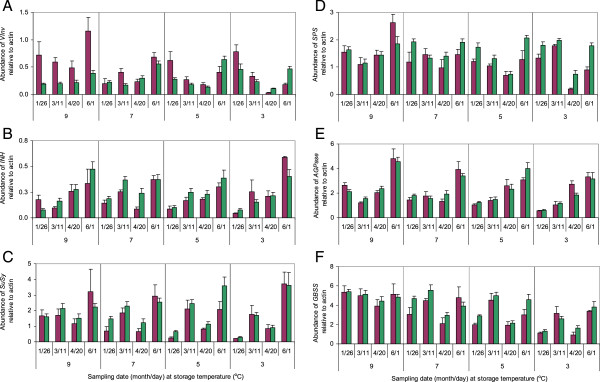
**Tuber transcript levels.** Transcript levels of vacuolar acid invertase (*VInv*, **A**), invertase inhibitor (*INH*, **B**), sucrose synthase (*SuSy*, **C**), sucrose phosphate synthase (*SPS*, **D**), ADP-glucose pyrophosphorylase small subunit (*AGPase*, **E**), and granule bound starch synthase (*GBSS*, **F**) in WT (purple bars) and RNAi (green bars) tubers relative to those of actin. Bars represent mean expression in twelve independent samples ± standard error. Tubers were harvested on October 1, 2010; stored at 3, 5, 7, or 9°C; and sampled in 2011.

Differences in *VInv* mRNA accumulation between RNAi and WT tubers were small and *VInv* mRNA content in RNAi lines was rarely less than one-third of that in WT lines (Figure 4A). All three RNAi lines had similar transcript levels, as did bud and stem ends of tubers, so their results were averaged to simplify presentation of the data. In tubers stored at 9°C, *VInv* mRNA abundance in RNAi tubers was 34% of that in WT tubers when averaged over all four sampling periods (Figure 4A). In tubers that were stored at lower temperatures, especially for longer times, there was less or no reduction of *VInv* message in RNAi tubers compared to WT tubers. Surprisingly, RNAi tubers stored at 3°C through April or June had more *VInv* mRNA than WT tubers.

Expression of the vacuolar invertase inhibitor gene *INH* was similar in RNAi and WT tubers at most times and temperatures (Figure 4B). Although significant differences for several samples were observed between *INH* mRNA accumulation in tubers of WT and RNAi lines, expression was not consistently higher in one or the other. In both WT and RNAi tubers at all storage temperatures, *INH* expression increased over time. Within each genotype and sampling time, there was no consistent change in *INH* expression with changing storage temperature.

The expression of *SuSy* and *SPS*, genes also involved in sucrose metabolism*,* was equal to or slightly higher in RNAi tubers than in WT tubers across sampling periods and storage temperatures with few exceptions. *SuSy* expression was higher in March and June than in January and April, especially at lower temperatures. Temperature- and time-dependent changes in gene expression were similar between RNAi tubers and WT. Although expression of *SPS* in RNAi tubers was statistically greater than in WT, especially at 3 and 5°C, the observed differences were less than 2-fold in almost all cases (Figure 4C,D).

For *AGPase* and *GBSS*, genes involved in starch synthesis, temperature- and time-dependent changes in gene expression were also observed (Figure 4E,F). Expression in January decreased with decreasing storage temperature for both of these genes, as it did for *SuSy* (Figure 4C). This decrease in expression with decreasing temperature persisted for *GBSS* at later times but not for *AGPase* or *SuSy*. At lower temperatures, *AGPase* expression increased over time, and *GBSS* expression, like that of *SuSy*, was higher in March and June than in April. Differences in mRNA accumulation between RNAi and WT tubers for *AGPase* and *GBSS* were relatively small or non-existent at almost all storage temperatures and times.

## Discussion

Suppression of vacuolar invertase gene expression in three lines of Katahdin effectively prevented reducing sugar accumulation through eight months of cold storage. Vacuolar invertase activity in RNAi tubers was 10-20% of that in WT tubers regardless of storage temperature or duration. Surprisingly, the amounts of *VInv* mRNA in RNAi tubers stored at 7°C or less were comparable to or greater than those in WT tubers during the later half of the storage period. In a previous study, expression of *VInv* in tubers of these highly suppressed RNAi lines was ≤3% of that in WT after 2 weeks of storage at 4°C [16]. At that time, the ratio of *VInv* expression to *actin* expression was 110 in WT tubers and between 1 and 4 in RNAi tubers. That study, however, did not investigate transcript levels later in storage. The data in Figure 4 show that *VInv* mRNA abundance in WT tubers late in storage was strongly reduced by endogenous regulatory mechanisms, and the ratio of *VInv* expression to *actin* expression was 1.5 or less in WT tubers from January through June. *VInv* expression in RNAi tubers was comparable to that in WT at that time. Thus, large differences in tuber reducing sugar content and invertase activity during late storage reflect the large differences in *VInv* mRNA abundance that occurred at an earlier time, rather than the small or negligible differences observed at later times. Since differences in invertase inhibitor activity between RNAi and WT tubers were not observed in the present study, persistent differences in invertase activity might reflect differences in invertase activity that were established early in the storage period and were maintained by stability of the invertase protein.

In Russet Burbank and Ranger Russet tubers with *VInv* expression effectively silenced by RNAi, differences in invertase activities and reducing sugar contents between WT and transgenic tubers were much larger than differences in *VInv* mRNA after three months of storage [22]. This observation is consistent with the data presented here for tubers from RNAi and WT Katahdin. Invertase expression increased approximately 6-fold at the tuber stem end and 15-fold at the tuber bud end in WT Russet Burbank between 1 and 5 months after harvest [22]. This pattern of gene expression is different from that observed for WT Katahdin, where *VInv* gene expression decreased or remained approximately constant until April and then increased modestly by 2- to 4-fold in June. An important area for future research will be to determine if this difference between cultivars can be generalized to a difference between round potatoes in the chip-processing market class and elongated potatoes in the fry-processing class.

RNAi tubers stored at 3-9°C had significantly greater sucrose accumulation at the stem ends than did WT tubers. Thus, vacuolar invertase activity in WT tubers helped to establish the sucrose set point for the tuber stem end. Bud-end sucrose content in RNAi tubers was similar to that in WT tubers at temperatures above 5°C. In RNAi tubers at 3-5°C, bud-end sucrose contents were greater than those in WT tubers but less than stem-end sucrose contents in RNAi tubers. Invertase activity was similar at the stem and bud ends of RNAi tubers (Figure 3). These observations suggest that sucrose may be compartmentalized differently in the two ends of the tubers, with more sucrose transported into or retained in the vacuole and accessible to VInv in cells at the tuber stem end than at the tuber bud end.

Reducing sugars in WT and RNAi tubers accumulated to higher levels at cooler temperatures (3 and 5°C) than at warmer temperatures (7 and 9°C). However, invertase activity tended to be greater at 7 and 9°C than at 3 and 5°C. Hence, differences in invertase activity between tubers stored under comparable conditions appear to correlate well with reducing sugar accumulation [30], but activity differences between different storage conditions or different developmental times may not correlate well with tuber reducing sugar content. Sugar content in both WT and RNAi tubers was responsive to different low temperature storage conditions, indicating that suppression of *VInv* did not eliminate temperature-dependent signaling pathways for sugar metabolism.

Glucose/fructose ratios were higher in the stem ends than the bud ends of WT tubers, suggesting that fructose is metabolized more rapidly than glucose in the tuber stem end. This difference could reflect differences in transport through the tonoplast or utilization of these sugars between the two ends of the tubers. For example, there may be spatial differences in the rate of glucose and fructose phosphorylation by cytosolic hexokinase and fructokinase, respectively [31, 32]. Ratios of glucose to fructose were similar at the two ends of RNAi tubers, where reducing sugar concentrations were lower than in WT. Differences between fructokinase and hexokinase activity are expected to be smaller at the stem end since substrate inhibition of fructokinase by fructose has less of an effect at low reducing sugar concentrations and hexokinases are not inhibited by glucose [31]. Interpretation of these data is complicated by an incomplete understanding of how reducing sugars are allocated between the cytosol, apoplast and vacuole in stored tubers.

Transcriptional regulation of several key enzymes in sucrose (*SuSy* and *SPS*) and starch (*AGPase* and *GBSS*) metabolism were investigated. Quantitative trait loci related to CIS have been linked to SuSy, SPS, and AGPase [33]. In both RNAi and WT tubers, mRNA accumulation of these genes varied depending on storage temperature and time in storage. However, the suppression of *VInv* expression by RNAi and the resulting large changes in sucrose, glucose and fructose contents had minor, if any, effects on the expression of these genes. In many cases, RNAi tubers contained twice as much sucrose as WT tubers, and WT tubers contained as much as 13 times the glucose and fructose of RNAi tubers, but differences in gene expression between WT and RNAi tubers were less than 2-fold in almost all cases. These data show that these genes are not subject to transcriptional regulation via sugar sensing or cross-talk with *VInv* expression. The fact that expression of these genes responded to temperature differences in the same way in WT and RNAi tubers indicates that they are likely involved in the temperature responsiveness of starch metabolism in stored tubers, which could also explain why RNAi tubers retained temperature sensitivity despite having reduced *VInv* expression and activity.

## Conclusions

*VIn*v suppression resulted in low VInv activity in Katahdin tubers stored for eight months, which led to large differences in tuber reducing sugar content and fried chip color between WT and RNAi tubers throughout the storage period. Low mRNA abundance and post-transcriptional control of VInv activity contributed to maintaining these differences through late storage. Though VInv expression and activity were reduced and sucrose, glucose, and fructose contents were very different in WT and RNAi tubers, expression of *AGPase* and *GBSS*, genes involved in starch metabolism, was largely unaffected by *VInv* silencing. Expression of *AGPase* and *GBSS* was, however, affected by storage temperature, as were tuber sugar contents. Taken together, these data indicate that the starch metabolism genes investigated here are not subject to regulation by sugar sensing or cross-talk with *VInv* expression, though they are regulated by storage temperature; the latter likely explains why RNAi tubers retained sensitivity to storage temperature despite having reduced *VInv* expression and activity. Additional evidence suggests that differential compartmentalization of sucrose occurs at the two ends of stored tubers.

## Abbreviations

AGPase: ADP-glucose pyrophosphorylase
CIPC: Isopropyl N-(3-chlorophenyl)carbamate
CIS: Cold-induced sweetening
cv.: Cultivar
FW: Fresh weight
GBSS: Granule-bound starch synthase
INH: Invertase inhibitor
L: Luminosity
RNAi: RNA interference
SPS: Sucrose phosphate synthase
SuSy: Sucrose synthase
VInv: Vacuolar acid invertase
WT: Wild-type.
